# Evaluation of alternately combining HPV viral load and 16/18 genotyping in secondary screening algorithms

**DOI:** 10.1371/journal.pone.0220200

**Published:** 2019-07-26

**Authors:** Hongxue Luo, Hui Du, Jerome L. Belinson, Ruifang Wu

**Affiliations:** 1 Department of Obstetrics and Gynecology, Peking University People’ Hospital, Beijing, PR China; 2 Department of Obstetrics and Gynecology, Peking University Shenzhen Hospital, Shenzhen, PR China; 3 Shenzhen Key Laboratory on Technology for Early Diagnosis of Major Gynecological diseases, Shenzhen, PR China; 4 Gynecologic Oncology Division, Women’s Health Institute, Cleveland Clinic, Cleveland, OH, United States of America; 5 Preventive Oncology International, Cleveland Heights, OH, United States of America; Bharathidasan University, INDIA

## Abstract

**Background:**

Correlation with HPV viral load and worsening cervical lesions had been reported, but its potential for triage after primary HPV screening has not been adequately explored, especially when combined with HPV-16/18 genotyping.

**Objective:**

To evaluate combinations of human papillomavirus (HPV) viral load and genotyping for HPV-16/18 as secondary screening strategies.

**Methods:**

The Shenzhen Cervical Cancer Screening Trial Ⅱ (SHENCCAST Ⅱ) database was re-analyzed to explore new screening algorithms using the results of Hybrid Capture 2 (HC2), Mass Array Matrix-Assisted Laser Desorption/Ionization Time-of-Flight Mass spectrometry System (MALDI-TOF-MS) and the ThinPrep cytologic test (TCT) obtained by endocervical sampling.

**Results:**

Compared with the recommended screening strategy of genotyping HPV-16/18 plus reflex to cytology, using viral load (10 RLU/CO as threshold) plus reflex to cytology resulted in less cytology but had a significantly higher sensitivity for cervical intraepithelial neoplasia 2+ (CIN2+)/CIN3+ without considerable changes in specificity and referral rates. Both of the strategy of using viral load ≥10 RLU/CO as cut-point for immediate colposcopy followed by triage genotyping HPV-16/18 for the other positive (≥1<10 RLU/CO) and the strategy of referring HPV-16/18 positives for immediate colposcopy followed by triage viral load (10 RLU/CO as threshold) for non-HPV-16/18 positives had comparable screening efficacy with algorithims that contain cytology.

**Conclusions:**

Primary HPV screening with triage of HPV-positive women by a combination of viral load and genotyping for HPV-16/18 provides good balance between sensitivity and specificity, the number of tests required, and referral rates.

## Background

Today there is consensus that persistent infection with high risk types of the human papillomavirus (hrHPV) is the essential cause for the development of cervical cancer[1, 2]. Primary hrHPV screening is now widely accepted based on evidence supporting increased detection and greater reassurance of a negative test than cytology based screening[3–6]. It also triggers the debate on triage strategies for the hrHPV-positive women since the most HPV infections are transient and harmless[7, 8]. Available options for triage should be based on the confirmed correlation with lesion grade. This can lead to a risk stratification using abnormal cervical cytology, P16 /Ki-67 dual-staining, HPV genotyping as well as some newer biomarkers[9–11]. However, no single current triage strategy, alone or in combination, has achieved the desired balance between screening effectiveness and economic burden.

In 2014, the interim guidelines from the Society of Gynecologic Oncology and the American Society for Colposcopy and Cervical Pathology recommended applying HPV genotyping plus reflex cytology[12]. This specific clinical approach was supported by the large Addressing THE Need for Advanced HPV Diagnostics HPV Study [13, 14]. In 2017 we reported the application of viral load (using the surrogate relative light units/control from HC2). We explored the significant correlation with worsening cervical lesions and introduced an algorithm for its application. We suggested using HPV viral load ≥10 RLU/CO to refer for immediate colposcopy with triage cytology for positives with ≥1<10 RLU/CO[15]. This analysis was drawn from the data obtained in the Shenzhen Cervical Cancer Screening Trial Ⅱ (SHENCCAST Ⅱ)[16–18]. Building on this prior work we believe the combination of HPV viral load and genotyping has not been adequately explored. Therefore, we decided to return to the SHENCCAST II database and analyze the range of algorithms focusing on physician-collected endocervical specimens tested by HC2 (Hybrid Capture 2) and MALDI-TOF-MS (matrix-assisted laser desorption/ionization time-of-flight mass spectrometry), a PCR-based multiplex genotyping assay.

## Objectives

The objective of this study was to evaluate the feasibility of alternately using viral load and genotyping for HPV-16 and/or HPV-18 (HPV-16/18) with or without cytology for triage after primary endocervical hrHPV testing.

## Study design

### 3.1 Study protocol

The SHENCCAST Ⅱ trial, which was conducted in 7 sites in Guangdong Province, China from 2009 to 2010, enrolled more than 10000 women ages 25 to 59 years, non-pregnant, with no previous history of pelvic radiation, a total hysterectomy, or cervical cancer screening within the previous 3 years. All participants signed an informed consent document before enrollment. The study protocol was approved by the institutional review boards from the Peking University Shenzhen Hospital, Shenzhen, China and the Cleveland Clinic, Cleveland, Ohio, USA. All participants contributed a self-collected sample for 2 HPV assays (MALDI-TOF-MS, Cervista) and a direct endocervical specimen for 3 HPV assays (HC2, MALDI-TOF-MS, Cervista) plus cytology (ThinPrep, TCT, Hologic Inc.). All women with abnormal cytology (atypical squamous cells of undetermined significance or worse, ≥ASC-US) or those testing hrHPV positive by self-sampling or direct sampling were referred for colposcopy and biopsy[16–18]. Cervical intraepithelial 2+ (CIN2+)/CIN3+ were used as endpoints to evaluate the performance of various screening strategies on the basis of the data obtained from endocervical specimen tested by HC2 and MALDI-TOF-MS.

### 3.2 Screening algorithms

Nine groups of algorithms using HPV viral load, 16/18 genotyping and cytology were evaluated.

**Group 1**—primary cytology, consists of 2 subgroups (1a, 1b) with referral for colposcopy all women ≥ASC-US or ≥LSIL.

**Group 2**—primary HPV, consists of 2 subgroups (2a, 2b), with referral for colposcopy all women testing positive: MALDI-TOF-MS any high risk sub-type (14 types) or HC2 ≥1 RLU/CO (13 pooled types).

**Group 3**—primary HPV with secondary cytology, consists of 2 subgroups (3a, 3b), with referral for colposcopy all women who are hrHPV positive (testing by HC2 or MALDI-TOF-MS) and ≥ASC-US. The women hrHPV positive with NILM are suggested 1yr F/U.

**Group 4—**primary viral load, consists of 4 subgroups (4a, 4b, 4c, 4d), with referral for colposcopy all women above the designated RLU/CO cut-points (10, 20, 50, 100). The ones below cut-point are suggested 1yr F/U.

**Group 5**—viral load plus reflex cytology, consists of 4 subgroups (5a, 5b, 5c, 5d), with referral for colposcopy all women ≥10 RLU/CO (or other cut-point options 20, 50, 100), then the women ≥1 RLU/CO < chosen cut-point have reflex cytology, and the ones ≥ASC-US are referred. The women ≥1 RLU/CO < chosen cut-point with NILM are suggested 1 yr F/U.

**Group 6**—primary HPV genotyping, with referral for colposcopy all women who are 16/18 positive. The women Non-16/18 positive are suggested 1yr F/U.

**Group 7**—genotyping plus reflex cytology, with referral for colposcopy all women who are 16/18 positive, and the women Non-16/18 positive have reflex cytology, and the ones ≥ASC-US are referred. The women Non-16/18 positive with NILM are suggested 1 yr F/U.

**Group 8**—viral load plus reflex genotyping, consists of 3 subgroups (8a, 8b, 8c), with referral for colposcopy all women ≥10 RLU/CO (or other cut-point options 20, 50), then the women ≥1 RLU/CO < chosen cut-point have reflex genotyping, and the ones 16/18 positive are referred.The women ≥1 RLU/CO < chosen cut-point with 16/18 negative are suggested 1 yr F/U.

**Group 9—**genotyping plus reflex viral load, consists of 3 subgroups (9a, 9b, 9c), with referral for colposcopy all womenwho are16/18 positive, then the women Non-16/18 positive have reflex viral load, and the ones ≥10 RLU/CO (or other cut-point options 20, 50) are referred. The women Non-16/18 positive with < chosen cut-point are suggested 1 yr F/U.

## Statistical analysis

The specificities for different algorithms were compared using the McNemar test on the subset of patients with negative biopsy finding. Likewise, in the comparisons of the sensitivities, the McNemar exact test was used on the patients with CIN2+/3+ finding for the subset is small. Confidence intervals were exact binomial confidence intervals (not shown), The difference were considered significant when p values of was less than .05. Data analyses were performed using STATA 14.0 (StataCorp LP, College Station, Tex). The reverse of positive predictive value (PPV) was equal to the number of colposcopies needed to detect 1 case of CIN2+/3+.

## Results

### 5.1 Cohort characteristics

The mean age of the 8556 cases eligible for this analysis was 38.9 years. The prevalence of CIN2+/3+ were 2.72%(233/8556)/1.65%(141/8556); Cytological abnormalities of ≥ASC-US/LSIL/HSIL were found in 12.1%/4.8%/1.4% of the study population; The direct endocervical specimens showed a proportion of 13.67%/8.87%/7.61%/6.21%/5.20% for HPV viral load of ≥1/10/20/50/100 RLU/CO by HC2 assay. Using the MALDI-TOF-MS assay we found a positivity of 11.93% and 2.77% for all high-risk HPV types and 16/18 subtypes respectively.

### 5.2 Evaluation of screening strategies

#### 5.2.1 Cytology or HPV testing alone

The primary cytology screening alone using ASC-US as threshold (Group 1a) showed a low sensitivity and a moderate specificity for CIN2+/3+ compared with primary HPV algorithm (Group 2) which had almost the highest sensitivity in all groups but referred many more people than others. Although Group 1b had a high specificity, the sensitivity was just 65.67% for CIN 2+ and 75.18% for CIN3+ (Table 1).

**Table 1:** Clinical outcomes of different screening algorithms for detection of CIN2 and CIN3 endpoint.

Screening algorithms	Colposcopy referral (%)	Cytology (%)	MALDI (%)	HC2 (%)	Colposcopies to detect 1, CIN2+/3+	CIN2+(n = 233)		CIN3+(n = 141)	
						Sensitivity(%)	Specificity(%)	Sensitivity(%)	Specificity(%)
**1. Cytology alone**									
*1a*. *ASC-US as threshold*	12.05	100.00	NA	NA	5.3/8.2	83.69	89.96	88.65	89.23
*1b*. *LSIL as threshold*	4.69	100.00	NA	NA	2.6/3.8	65.67	97.02	75.18	96.49
**2. HPV alone**									
*2a*. *HC2 (rlu/co = 1 as cutoff)*	13.67	NA	NA	100.00	5.2/9.1	95.71	88.62	97.87	87.74
*2b*. *MALDI (Genotyping)*	11.93	NA	100.00	NA	4.7/7.7	92.70	90.33	94.33	89.45
**3. Primary HPV with secondary cytology**									
*3a*. *HC2 (rlu/co = 1 as cutoff)*	6.60	13.67	NA	100.00	3.0/4.6	81.55	95.49	87.23	94.75
*3b*. *MALDI (Genotyping)*	6.11	11.93	100.00	NA	2.8/4.4	78.97	95.93	83.69	95.19
**4. Viral load alone**									
*4a*. *(rlu/co = 10 as cutoff)*	8.87	NA	NA	100.00	3.7/5.8	88.84	93.37	92.20	92.53
*4b*. *(rlu/co = 20 as cutoff)*	7.61	NA	NA	100.00	3.3/5.2	84.55	94.55	88.65	93.75
*4c*. *(rlu/co = 50 as cutoff)*	6.21	NA	NA	100.00	2.9/4.6	78.11	95.81	82.27	95.07
*4d*. *(rlu/co = 100 as cut off)*	5.20	NA	NA	100.00	2.9/4.5	65.24	96.48	65.56	95.09
**5. Viral load plus reflex cytology**									
*5a*. *(rlu/co = 10 triage)*	10.00	4.80	NA	100.00	3.9/6.3	93.13	92.32	96.45	91.44
*5b*. *(rlu/co = 20 triage)*	9.13	6.07	NA	100.00	3.6/5.8	91.85	93.19	95.04	92.31
*5c*. *(rlu/co = 50 triage)*	8.39	7.47	NA	100.00	3.4/5.4	90.13	93.90	93.62	93.04
*5d*. *(rlu/co = 100 triage)*	8.00	8.47	NA	100.00	3.4/5.3	86.70	94.24	91.49	93.44
**6. Genotyping (HPV-16/18)**	2.77	NA	100.00	NA	2.5/3.2	41.20	98.36	53.19	98.07
**7. Genotyping plus reflex cytology**	7.23	9.16	100.00	NA	3.1/4.8	84.55	94.93	90.78	94.17
**8. Viral load plus reflex genotyping**									
*8a*. *(rlu/co = 10 triage)*	9.29	NA	4.80	100.00	3.7/5.9	92.27	93.03	95.04	92.14
*8b*. *(rlu/co = 20 triage)*	8.18	NA	6.07	100.00	3.4/5.3	89.27	94.09	92.91	93.24
*8c*. *(rlu/co = 50 triage)*	6.95	NA	7.47	100.00	3.3/4.8	84.12	95.21	87.94	94.40
**9. Genotyping plus reflex Viral load**									
*9a*. *(rlu/co = 10 triage)*	8.94	NA	100.00	9.16	3.6/5.9	90.13	93.33	92.20	92.45
*9b*. *(rlu/co = 20 triage)*	8.13	NA	100.00	9.16	3.4/5.5	87.12	94.08	90.07	93.24
*9c*. *(rlu/co = 50 triage)*	7.09	NA	100.00	9.16	3.1/5.0	82.83	95.03	85.82	94.22

ASC-US indicates atypical squamous cells of undetermined significance; LSIL indicates low grade squamous intraepithelial lesion

CIN, cervical intraepithelial neoplasia; HC2, Hybrid Capture 2; MALDI, Mass Array Matrix-Assisted Laser Desorption/Ionization Time-of-Flight Mass spectrometry System; HPV, humanpapillomavirus; NA, not applicable; rlu/co, relative light units/control.

#### 5.2.2 Primary HPV testing and secondary cytology

If HPV testing was the primary screening with secondary cytology, then only the HPV positive cases with cytology ≥ASC-US were referred (Group 3). This algorithm would reduce the referral rate to 6.60% and 6.11% (HC2 and MALDI), but results in a much lower sensitivity compared with the algorithm of primary HPV testing (Group 2) (Table 1).

#### 5.2.3 HPV viral load or genotyping alone

Increasing the cut-point of the HC2 assay in Group 4 could improve the specificity and decrease the referral rate, but the cost as expected is a decrease in sensitivity. When 10 or 20 RLU/CO was used as the cut-point for viral load, the corresponding algorithms (Group 4a & 4b) had a comparable sensitivity for CIN2+/3+ using primary cytology (Group 1), but resulted in a significantly lower referral rate (8.87% & 7.61% vs. 12.05%, p< 0.05) (Table 1).

The algorithm of just referring HPV16 and/or 18 positives to colposcopy (Group 6) had the highest specificity, but the sensitivity for CIN2+ and CIN3+ were only 41.20% and 53.19% respectively (Table 1).

#### 5.2.4 Primary HPV viral load with secondary cytology or HPV genotyping

The algorithm viral load plus reflex cytology (Group 5a) used viral load 10 RLU/CO as the cut-point for immediate colposcopy and then triaged the other HPV positives (≥1<10 RLU/CO) using cytology. This model only added 4.80% cytology testing, but reached an sensitivity of 93.13%/96.45% and specificity of 92.32%/91.44% for CIN2+/3+, as well as referral rates (10.00%). By increasing the HPV positive cut-point all values changed; 10 RLU/CO seemed to be the most acceptable point to use (Table 1).

If cytology was eliminated and HPV-16/18 genotyping was used to triage primary HPV viral load screening directly (Group 8), a new balance between sensitivity and specificity could be reached. When viral load 10 RLU/CO was defined as the cut-point (Group 8a), no significant difference could be found on both sensitivity and specificity compared with Group 5a. Improving the cut-point could reduce the referral rate but again at the cost of decreased sensitivity (Table 1).

#### 5.2.5 Primary HPV genotyping with secondary cytology or HPV viral load

Group 7 used cytology as triage for Non-16/18 HPV positives. Though adding 9.16% more cytology testing, this algorithm could dramatically improve the sensitivity for CIN2+/3+ (84.55%/90.78% vs. 41.20%/53.19%, p<0.01/p<0.01) compared to primary HPV-16/18 genotyping alone (Group 6). However, compared to the algorithm of viral load followed by cytology (Group 5a), Group 7 was less sensitive, especially for CIN2+ (93.13% vs. 84.55%, p<0.01), although it reduced the number of colposcopies (Table 1).

If when primary HPV genotyping was performed, and viral load level was used to triage the Non-16/18 positive cases (Group 9), a comparable result of sensitivity for CIN2+/3+ would be seen not only with Group 7 (90.13%/92.20% vs. 84.55%/90.78%, p>0.05/p>0.05) but also with Group 5a (90.13%/92.20% vs. 92.27%/95.04%, p>0.05/p>0.05). Raising the cut-point for viral load (positive) would produce a new balance with decreasing sensitivity and an increasing specificity (Table 1).

## Discussion

Primary high-risk HPV screening is now widely accepted, but the switch from primary cytology to a primary HPV test has evolved over many years. The newer HPV genotyping technologies have added a new element to screening. HPV genotyping can be used for risk stratification in primary screening based on the correlation between different HPV subtype infections and cervical lesions, making it practical for combinations with an additional triage technology, such as cytology, to reach a balance between safety and test utilization appropriately.

HC2 performs HPV testing and reports an HPV viral load surrogate simultaneously, and the viral load level is known to have positive association with the grade of cervical lesions[19–21]. Our previous manuscript reported the advantage of stratifying viral load then using cytology to triage the low viral load group compared with primary cytology or HPV testing. Here, we have refined our analysis by changing the threshold of the semi-quantitative viral load. Our analytic results showed that the algorithm using viral load 10 RLU/CO as the cut-point (Group 5a) had the largest impact on sensitivity for CIN2+/3+ compared with the recommended algorithm, and even if the cut-point was set at 100 RLU/CO, all indices were still comparable.

In view of the highest specificity of Group 6, we tried to use HPV-16/18 genotyping to triage the HPV positives with relatively low viral load instead of cytology in Group 5 (Group 8). When 10 RLU/CO was chosen as the cut-point, the sensitivity of such an algorithm (Group 8a) was significantly higher than the recommended algorithm, and was comparable with the algorithm viral load (10 RLU/CO as cut-point) plus reflex cytology (Group 5a). Because of increasing the cut-point of viral load (Group 4) could lead to a better specificity than cytology, we then tried to change cytology of the recommended algorithm (Group 7) to viral load detection to triage those Non-16/18 HPV positives (Group 9). Different RLU/CO values were analyzed, but only by using 10 RLU/CO as the cut-point (Group 9a), could such an algorithm produce a significantly higher sensitivity compared with Group 7.

The prevalence of high-risk HPV types vary geographically. In parts of Asia we know that HPV-31/33/52/58 are reported as frequently as HPV-16 or 18 in pre-cancers[22–24]. Whether or not the recommended algorithm could be refined further by recognizing the importance of other high-risk types is worth consideration. However it is important to recognize that the closer the diagnosis is to cancer, the greater the importance of HPV-16/18 world-wide. Additionally, the impact of HPV vaccination in future will significantly impact future screening depending on the HPV types included in the applied vaccines and their coverage in individual populations.

In Group 8 and Group 9, we alternately combined HPV viral load and HPV-16/18 genotyping and obtained a comparable secondary screening method without the recommended cytology. With this in mind, if the PCR based Cobas 4800 HPV test or similar assays had the capacity of outputting the data for viral load, a more efficient algorithm could easily be developed. If one HPV test could display the results of viral load and genotyping simultaneously, not only the cost could be reduced, but other triage methods could easily be combined.

It is worth mentioning that, the difference to number of colposcopies needed to detect one CIN2+/CIN3+ was not considerably different (3.7/5.9 vs. 3.6/5.9) between Group 8a and Group 9a. However, between Group 4a and Group 6, it was 3.7/5.8 vs. 2.5/3.2, which reminded us the cutoff 10 RLU/CO might be unapplicable to triage all non-16/18 positives for its ruling out some type-specific ones which could cause high grade lesions in low viral load level. Therefore, grouping viral load according to different types and estimating their risks to reach CIN2+/3+ would refine the algorithms. Some studies reported P16/Ki-67 and methylation markers had better sensitivity and specificity than traditional cytology[25, 26], making them potential replacements to triage the Non-16/18 positives and low viral load groups in primary HPV genotyping and primary HPV viral load screening.

Another point of consideration in our current analysis was that all groups of algorithms contained the screening step of one year follow-up, but no data was available in the SHENCCAST Ⅱ trial data to assess the viability of this option. Therefore whether a strategy would perform the same over a lifetime in regions with repeated screening needs further research. However, it is important to recognize the great difficulty in achieving follow-up in many developing countries, especially in those remote areas, where the women might have the only one opportunity to receive screening.

In conclusion, the current analysis of screening algorithms based on the SHENCCAST Ⅱ trial demonstrated that viral load appears to have an undeveloped potential for triage after primary HPV screening especially when combined with HPV-16/18 genotyping or cytology. Hopefully in the future the bio-informatic phase of the new PCR based assays can be designed to include viral load in their output.
